# T cell response and cytokines production after allergen stimulation in children allergic to cow’s milk protein

**DOI:** 10.1186/2045-7022-3-S3-P47

**Published:** 2013-07-25

**Authors:** F Angelini, V Pacciani, S Corrente, E Monteferrario, ML Romiti, R Silenzi, V Moschese, L Chini

**Affiliations:** 1 Division of Pediatrics, University of Rome Tor Vergata, Rome, Italy; 2 Immunology, Allergology and Rheumatology Unit, Department of Pediatrics, Centre Hospitalier Universitaire Vaudois (CHUV), Lausanne, Switzerland; 3 Department of Pediatrics, Children's Hospital Bambino Gesù, Rome, Italy; 4 Division of Pediatrics, Allergy and Immunology Unit, University Hospital of Tor Vergata, University of Rome Tor Vergata, Rome, Italy

## Background

Cow’s milk protein (CMP) allergy (CMPA) affects 2-6% of children. The most important allergens are a-casein (ACA), a-lactoalbumin (ALA) and b-lactoglobulin (BLG). BLG, absent in human milk, is considered to be the major allergen of CMP. Little is known about T cells response and cytokines profile in relation to CMPA and tolerance acquisition. Our aim was to characterize T cells response and cytokines production to CMP in children with CMPA and in children who outgrew CMPA.

## Methods

Twenty-two children, Group I (n=13) and Group II (n=9), divided according to age (<3, 3-6 and >6 years), and 15 age-matched healthy controls (HC), were enrolled. T cells proliferation to CMP was measured in PBMC by 3H-thymidine incorporation. Responder was defined any patient displaying a stimulation index (SI) ≥mean+2SD SI of HC stimulated T cells (cut off for ALA, BLG, ACA = 2.5, 2.4 and 1.7, respectively). To study cytokine profile, T cell lines were generated by stimulation of PBMC of patients for 14 days with CMP in the presence of IL-2. Th1 (IFN-g, IL-2), Th2 (IL-4, IL-5) and Th17 (IL-17) cells were detected at day 0 and 14, after stimulation with PMA and Ionomycine for 5 h, by intracellular staining and flow cytometry.

## Results

T cell response to ACA was >cut off only in 5 children (mean SI 3.4+ 2.2, range 2-7.3), whereas proliferation to ALA (mean SI 10.8+ 14.4, range 2.7-49.6) and BLG (mean SI 16.8+ 22.1, range 2.5-76.3) was > cut off in the majority of them. Of note, in children aged 3-6 yrs, it was significantly greater in Group I than in Group II, (ALA *p*=0.04; BLG *p*=0.0034). Moreover, in Group I, proliferation to ALA and BLG showed a significant difference between non-IgE mediated and IgE-mediated CMPA (*p*=0.00023 and *p*=0.0038, respectively). Th1 and Th2 cytokines profile before and after CMP stimulation was similar in both groups, but in Group I Th17 cells were significantly increased after 14 days of culture with CMP (*p*=0.0008).

## Conclusion

Our preliminary results indicate a correlation between T cell response to CMP and immune mechanism (IgE-mediated and non-IgE mediated CMPA), allergic manifestation and symptom remission and suggest to implement *in vitro* T cell investigations in children with CMPA since they might constitute useful diagnostic markers of allergy and tools to follow up tolerance acquisition. Moreover, the increase of Th17 frequency in children with CMPA, suggest a role of Th17 cells in pathogenesis of food allergy.

## Disclosure of interest

None declared.

